# ^1^H, ^13^C and ^15^N NMR chemical shift assignments of cAMP-regulated phosphoprotein-19 and -16 (ARPP-19 and ARPP-16)

**DOI:** 10.1007/s12104-020-09951-w

**Published:** 2020-05-28

**Authors:** Chandan J. Thapa, Tatu Haataja, Ulla Pentikäinen, Perttu Permi

**Affiliations:** 1 Department of Biological and Environmental Science, University of Jyvaskyla, Jyvaskyla, Finland; 2 Institute of Biomedicine, University of Turku, Turku, Finland; 3 Turku Bioscience, University of Turku and Åbo Akademi, Turku, Finland; 4 Department of Chemistry, Nanoscience Center, University of Jyvaskyla, Jyvaskyla, Finland

**Keywords:** Assignments, cAMP-regulated phosphoprotein-19, HA-detection, intrinsically disordered protein, NMR spectroscopy

## Abstract

Protein Phosphatase 2A, PP2A, the principal Serine/threonine phosphatase, has major roles in broad range of signaling pathways that include regulation of cell cycle, cell proliferation and neuronal signaling. The loss of function of PP2A is linked with many human diseases, like cancer and neurodegenerative disorders. Protein phosphatase 2A (PP2A) functions as tumor suppressor and its tumor suppressor activity is inhibited by the overexpression of PP2A inhibitor proteins in most of the cancers. ARPP-19/ARPP-16 has been identified as one of the potential PP2A inhibitor proteins. Here, we report the resonance assignment of backbone ^1^H, ^13^C and ^15^N atoms of human ARPP-19 and ARPP-16 proteins. These chemical shift values can provide valuable information for the further study of the dynamics and interaction of ARPP-proteins to PP2A using NMR spectroscopy.

**Electronic supplementary material:**

The online version of this article (doi:10.1007/s12104-020-09951-w) contains supplementary material, which is available to authorized users.

## Biological context

cAMP regulated phosphoprotein-19 (ARPP-19), and its splice variant ARPP-16, were originally identified as substrates of the cAMP dependent protein kinase in neostriatum. ARPP-16 is highly expressed in neuronal cells, whereas, ARPP-19 is expressed ubiquitously (Horiuchi et al. 1990). In neuronal cells, ARPP-19 acts as bridge between nerve growth factor and post-transcriptional regulation of neuronal gene expression and controls the neuronal development and plasticity (Irwin et al. 2002). The reduced expression of ARPP-19 is associated with neurological disorders like Alzheimer’s disease and Down’s syndrome (Kim et al. 2001).

ARPP-19 and ARPP-16 also plays roles in the regulation of cell cycle. Recent studies have reported the role of ARPP-19 in the development and progression of several human cancer types, such as, breast cancer (Lü et al. 2015), hepatocellular carcinoma (Song et al. 2014) and human glioma (Jiang et al. 2016). Phosphorylation of ARPP-19 by the greatwall kinase (Gwl) promotes mitotic entry and maintenance of mitotic state by inhibiting PP2A (Andrade et al. 2017; Gharbi-Ayachi et al. 2010; Song et al. 2014). ARPPs phosphorylated by the MAST3 (Microtubule Associated Ser/Thr kinase 3) kinase, a homolog of MASTL/Gwl kinase, selectively inhibits tumor suppressor PP2A holoenzyme containing B55α and B56δ (Andrade et al. 2017).

Structural level characterization of ARPPs has remained elusive; thus far there is no published structural data available for either ARPP-19 or ARPP-16. Consequently, the interaction of ARPPs with their different binding partners have only been observed at low resolution. In this paper, we report the backbone assignment of ARPP-19 and ARPP-16 as a first step towards structural level understanding of ARPPs function.

## Methods and experiments

### Recombinant protein production and purification

The human cAMP regulated phosphoproteins, ARPP-16 and ARPP-19, were overexpressed in the *Escherichia coli* strain BL21 Gold from a plasmid vector carrying the gene conferring ampicillin resistance. For the production of uniformly ^13^C and ^15^N labelled ARPPs, cells were grown in standard M9 minimal media supplemented with 1 g/l of ^15^NH_4_Cl and 2 g/l ^13^C-d-glucose as the only nitrogen and carbon source, respectively. The proteins were produced as Glutathione S-transferase (GST) fusion proteins. *Escherichia coli* BL21 Gold cells with ARPP-19/ARPP-16 plasmids were cultured in M9 minimal containing 100 µg/ml ampicillin at 37 °C, shaking the culture at 250 rpm until the OD at 600 nm was 0.6. The cells were cooled down to 25 °C and expression of GST fusion proteins were induced by adding 0.4 mM isopropyl β-d-1-thiogalactose at 25 °C for 20 h, shaking the culture at 250 rpm.

The cells were lysed using EmulsiFlex-C3 homogenizer (Avestin) and subsequently centrifuged at 35,000×*g* for 30 min. The lysates were purified with Protino Glutathione Agarose 4B (Macherey-Nagel) according to the manufacturer’s instruction. GST was cleaved by Tobacco Etch Virus (TEV) protease (Invitrogen, Life Technologies) at 4 °C for 16 h and removed from the solution with the Glutathione Agarose. The proteins were further purified by size exclusion chromatography with a HiLoad 26/60 Superdex 200 pg column (GE Healthcare) in 50 mM NaH_2_PO_4_ pH 6.5, 100 mM KCl, 1 mM DTT using an ÄKTA pure chromatography system (GE Healthcare). Finally, the proteins were concentrated with Amicon ultra centrifugal 3K filter device (Millipore).

### NMR spectroscopy

NMR spectra of ARPP-19 and ARPP-16 were acquired at 298 K using a Bruker Avance III HD 800 MHz spectrometer equipped with a 5-mm ^1^H, ^13^C, ^15^N triple resonance TCI CryoProbe. All NMR spectra were measured in 95/5% 50 mM NaH_2_PO_4_, 100 mM NaCl, 1 mM DTT pH 6.5/D_2_O buffer. The concentration of ARPP-19 and ARPP-16 used in the data collection were 0.7 mM and 0.4 mM, respectively. For the sequence specific backbone and partial side chain assignment of ARPPs, we used a set of HN-detected triple resonance experiments i.e. HN(CO)CACB, HNCACB (Yamazaki et al. 1994), HNCO (Muhandiram and Kay 1994), i(HCA)CO(CA)NH (Mäntylahti et al. 2009) as well as HA-detected experiments iHA(CA)NCO, HA(CA)CON (Mäntylahti et al. 2010), and HA(CA)CON(CA)HA (Mäntylahti et al. 2011). NMR data were processed using TopSpin 3.5 software package (Bruker Corporation) and analysed using Sparky 3.13 (Lee et al. 2015).

### Assignment and data deposition

**Fig. 1:**
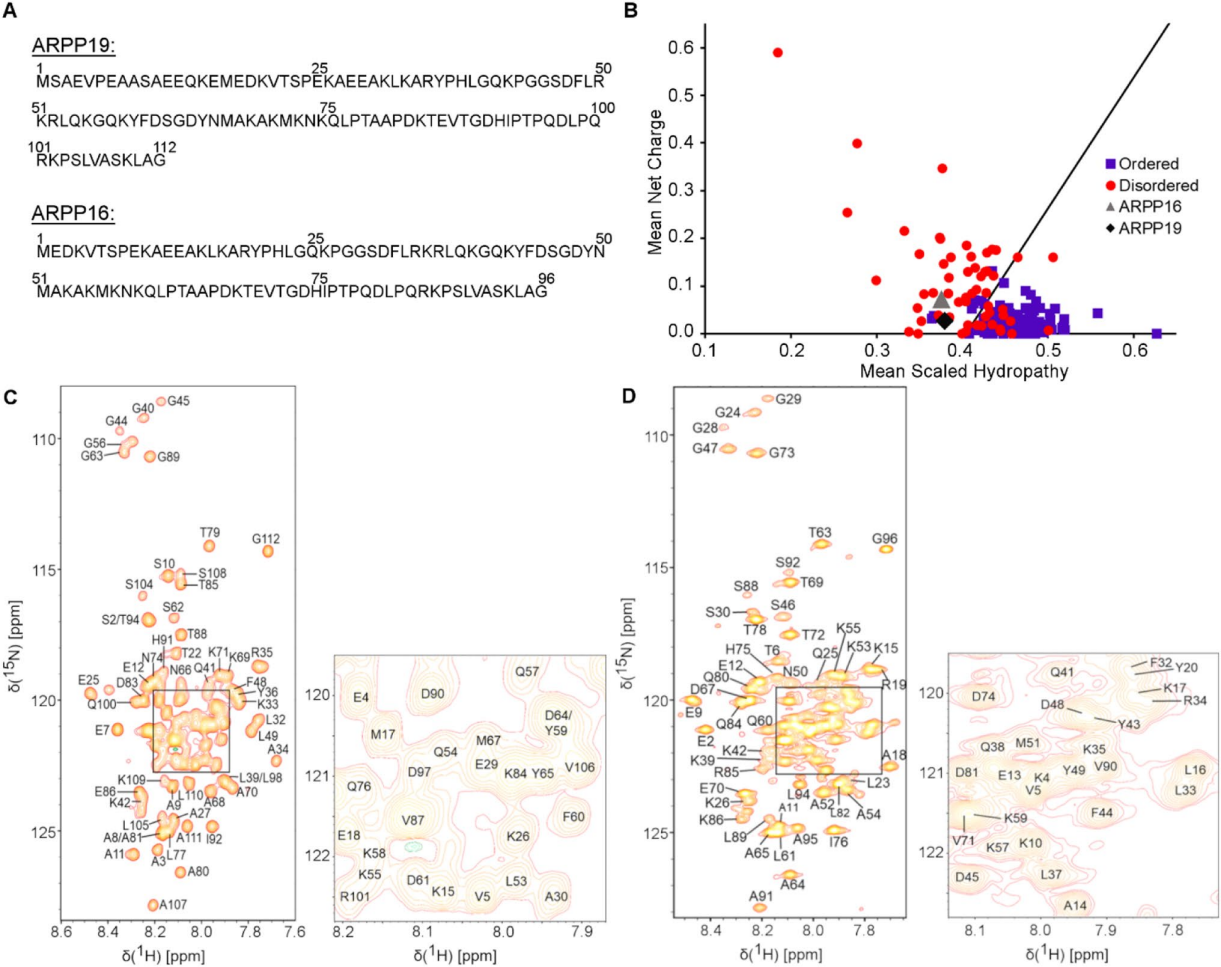
**a** The amino acid sequence of human ARPP-19 and ARPP-16. **b** Charge-hydropathy plot of human ARPP-19 and ARPP-16. The sequences of ARPP-19 and ARPP-16 were analyzed at http://www.pondr.com. The position of ARPP-19 and ARPP-16 are shown by green diamond and gray triangle, respectively, in comparison to a set of disordered (red circles) and ordered (blue squares) proteins. The border line drawn between disordered and ordered space is empirically defined by the equation *< H >* _*b*_*= (< R > +* 1.151)/2.785 (Uversky et al. 2000). **c** and **d **^1^H, ^15^N HSQC spectrum of ARPP19 and ARPP16, respectively. The NH resonances are labelled with *one-letter-amino-acid-codes* and residue numbers. Insets show the enlargement of the crowded NH regions in ARPPs

**Fig. 2:**
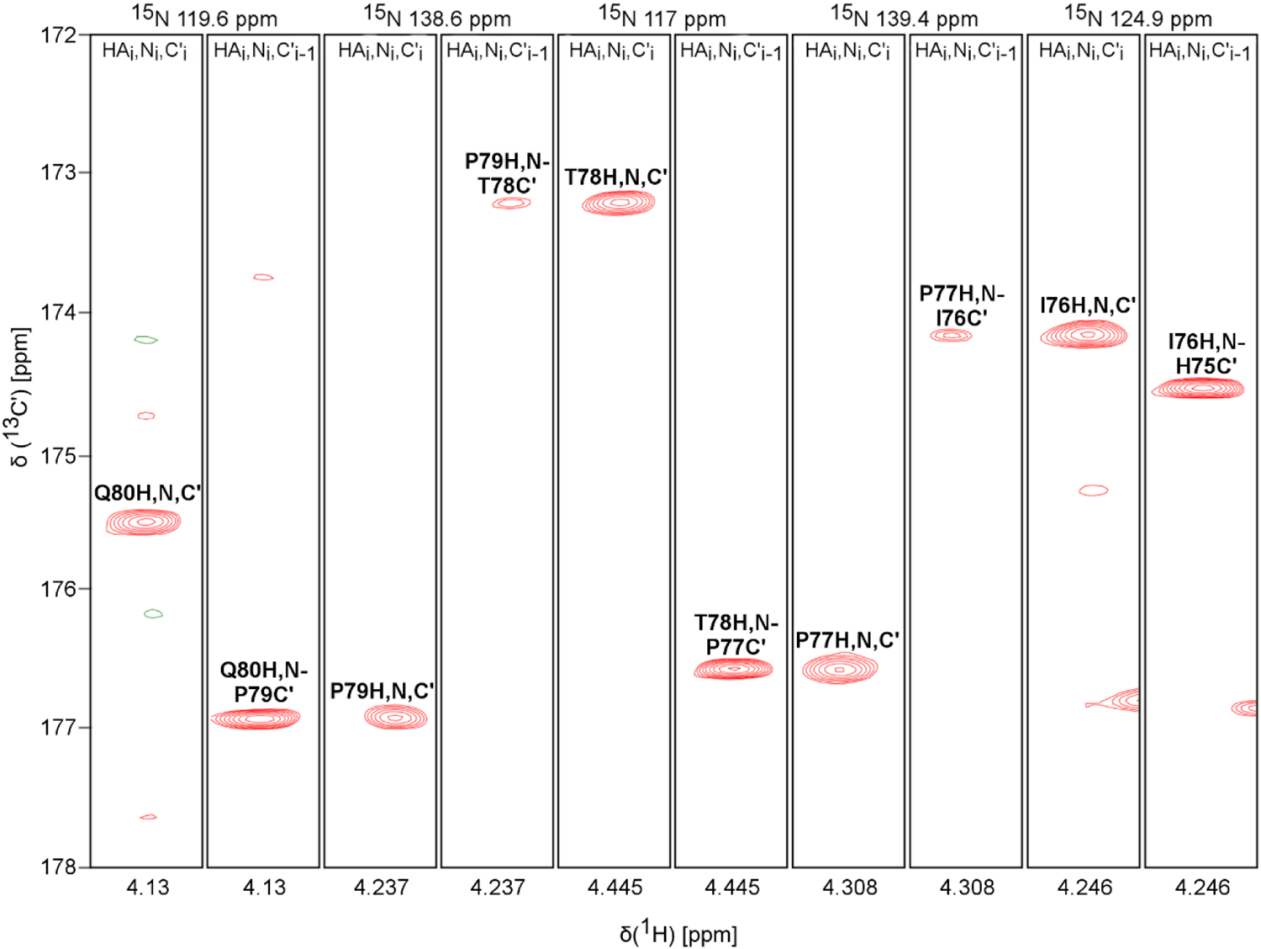
Sequential walk through prolines of ARPP-16. Assignment of the residues His75-Ile-Pro-Thr-Pro-Gln80 using iHA(CA)NCO and (HACA)CON(CA)HA spectra

**Fig. 3:**
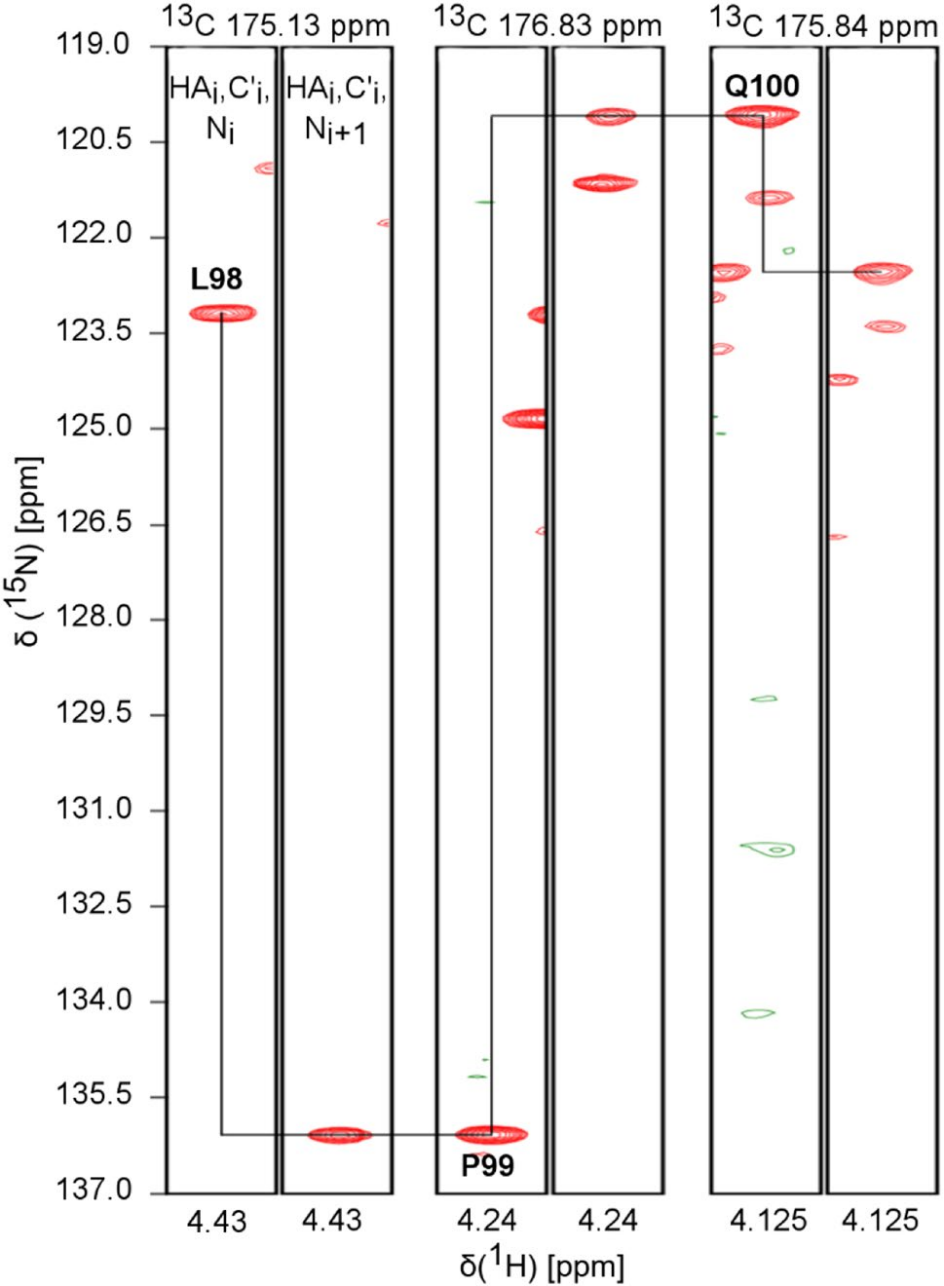
Sequential walk through proline of ARPP-19. Assignment of residues Leu98-Pro-Gln100 by employing iHA(CA)NCO and HA(CA)CON spectra

The amino acid sequence of ARPP-19 and ARPP-16 are shown in Fig. 1a. The examination of the amino acid sequence shows the enrichment of hydrophilic (26.2%) and charged (33%) residues as well as prolines (8.9%), basic features of intrinsically disordered proteins (IDPs) (Hazy and Tompa 2009; Uversky 2010). The predictor of natively unfolded protein VL-XT algorithm (Romero et al. 2001) indicates both ARPPs are 50% disordered, and the Uversky plot (Uversky et al. 2000) of mean charge against mean scaled hydropathy positions ARPPs among the set of intrinsically disordered proteins (Fig. 1b). The bioinformatical analyses were experimentally confirmed in ^1^H–^15^N 2D HSQC spectra of ARPPs, that is, the disordered nature of ARPPs manifests itself as low dispersion of chemical shift in the ^1^H^N^ dimension, all amide proton resonances falling between 7.7 and 8.5 ppm (Fig. 1c and d). Analogously, severe clustering of Cα and Cβ shifts in HNCACB/HN(CO)CACB spectra was observed, rendering backbone assignment based on Cα and Cβ shifts inefficient. We then resorted to the CO shift based assignment as a remedy i.e. employing HN-detected HNCO and its intraresidual counterpart i(HCA)CO(CA)NH experiments, and supplementing these data with HA-detected CO–N correlation experiments i.e. iHA(CA)NCO, HA(CA)CON, and (HACA)CON(CA)HA. By using the HA-detected spectra we were able to complete the backbone assignment passing the proline rich region uninterrupted. Representative illustrations of sequential walk utilizing iHA(CA)NCO and (HACA)CON(CA)HA, and iHA(CA)NCO and HA(CA)CON spectra for ARPP-16 and ARPP-19, respectively, are shown in Figs. 2 and 3. In this way, omitting N-terminal residues remaining after TEV cleavage, nearly complete backbone assignment of ARPP-19 and ARPP-16 was obtained. The ^1^H, ^15^N and ^13^C resonances of ARPP-16 and ARPP-19 have been deposited into BioMagResBank (http://www.bmrb.wisc.edu/) under accession numbers 27911 and 27912, respectively.

In ARPP-16, 99% of ^1^H-^15^N pairs (86 out of 87), 98% of ^13^Cα (94 out of 96), 99% of ^13^Cβ (88 out of 89), 97% of ^1^Hα (93 out of 96) and 100% of ^13^CO (96 out of 96) resonances were assigned. The ^1^H^N^ of the first residue, Met1, could not be assigned due to exchange broadening. In addition, Cα and Cβ shifts of Ser30 could not be assigned, because of mutual cancellation of opposite phased Cα and Cβ cross peaks. The absence of ^1^Hα resonances of Ser7, Arg36 and Gly40 are attributed to significant line broadening.

Similarly, 99% of ^1^H-^15^N pairs (101 out of 102), 98% of ^13^Cα (110 out of 112), 98% of ^13^Cβ (103 out of 105), 96% of ^1^Hα (108 out of 112) and 100% of ^13^CO (112 out of 112) resonances were assigned for ARPP-19. ^1^H^N^ of Ser46 could not be assigned probably due to fast exchange of amide proton with the solvent. Moreover, Cα and Cβ shifts of Glu13 and Lys102 could not be assigned from ARPP-19 as the resonance peaks are most likely overlapped and very weak. The missing ^1^Hα resonance of Gln14, which is overlapped with resonance of Lys75, prevents unambiguous assignment. The ^1^Hα resonances of Ser23, Arg52 and Gly56, which corresponds to the Ser7, Arg36 and Gly40 residues of ARPP16, are missing due to exchange broadening.

In this paper, we have presented a nearly complete assignment of main-chain ^1^H, ^13^C, and ^15^N chemical shifts in two intrinsically disordered proteins, ARPP-16 and ARPP-19. These assignments allow residue-level characterization of ARPP-16/19 dynamics and interactions in ongoing studies.

## Electronic supplementary material

Below is the link to the electronic supplementary material.

Electronic supplementary material 1 (PNG 105 kb)

Electronic supplementary material 2 (PNG 41 kb)

Electronic supplementary material 3 (PNG 32 kb)
