# Supplementary figures and images for: 1H, 13C and 15N NMR chemical shift assignments of cAMP-regulated phosphoprotein-19 and -16 (ARPP-19 and ARPP-16)
Source: Biomol NMR Assign. 2020 May 28;14(2):227–31. doi: 10.1007/s12104-020-09951-w (PMC7462833; doi:10.1007/s12104-020-09951-w)

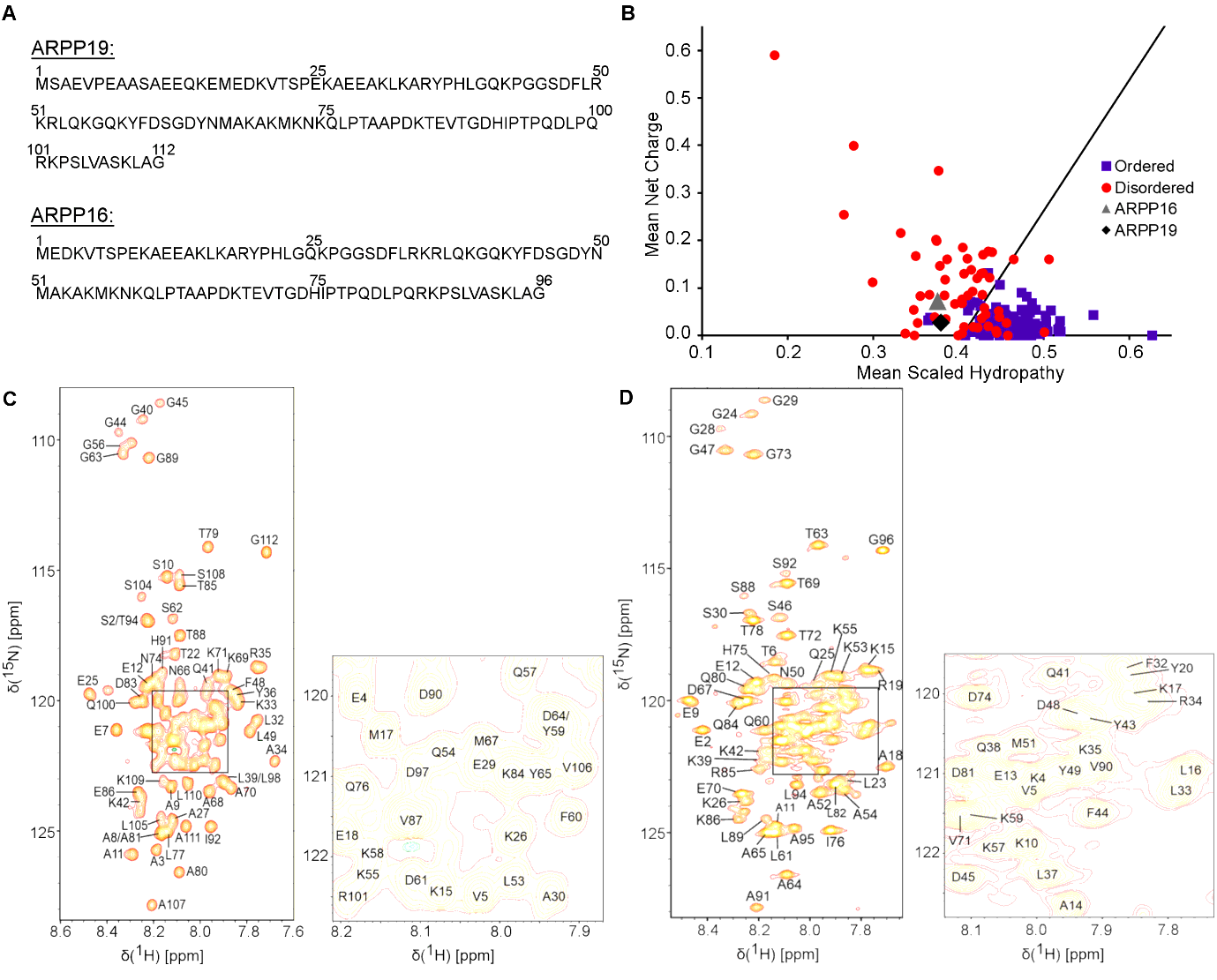

Supplement: Supplementary file 1 — Electronic supplementary material 1 (PNG 105 kb) [file 12104_2020_9951_MOESM1_ESM.png]

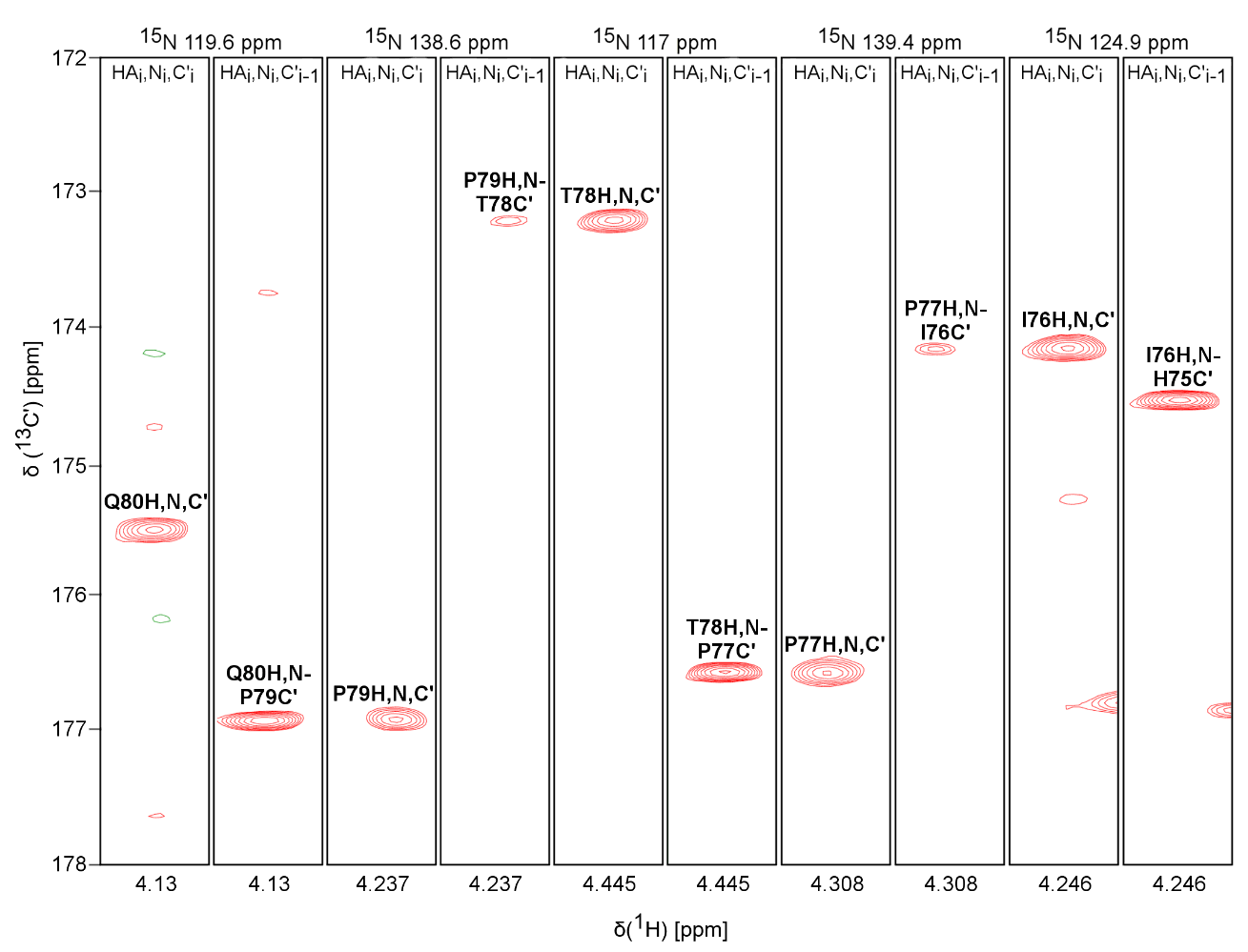

Supplement: Supplementary file 2 — Electronic supplementary material 2 (PNG 41 kb) [file 12104_2020_9951_MOESM2_ESM.png]

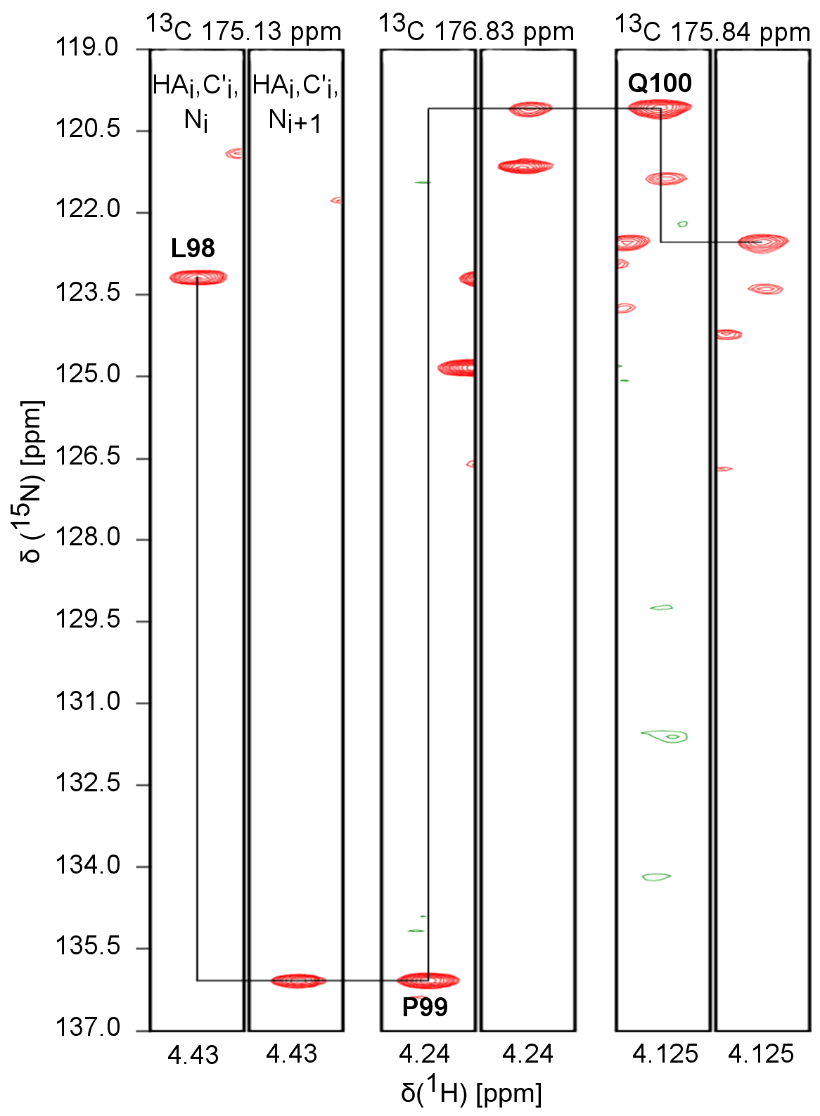

Supplement: Supplementary file 3 — Electronic supplementary material 3 (PNG 32 kb) [file 12104_2020_9951_MOESM3_ESM.png]
